# Factors Influencing Chinese Male's Willingness to Undergo Circumcision: A Cross-Sectional Study in Western China

**DOI:** 10.1371/journal.pone.0030198

**Published:** 2012-01-12

**Authors:** Xiaobo Yang, Abu S. Abdullah, Bo Wei, Junjun Jiang, Wei Deng, Bo Qin, Weili Yan, Qianqiu Wang, Chaohui Zhong, Qian Wang, Yuhua Ruan, Yunfeng Zou, Peiyan Xie, Fumei Wei, Na Xu, Hao Liang

**Affiliations:** 1 School of Public Health & Guangxi Key Laboratory Cultivation Base of AIDS Prevention and Treatment, Guangxi Medical University, Guangxi, China; 2 Department of Epidemiology, Robert Stempel School of Public Health, Florida International University, Miami, Florida, United States of America; 3 The First Affiliated Hospital, Chongqing Medical University, Chongqing, China; 4 School of Public Health, Xinjiang Medical University, Xinjiang, China; 5 School of Public Health, Chongqing Medical University, Chongqing, China; 6 National Center for STD Control, China Centers for Disease Control and Prevention, Nanjing, China; 7 State Key Laboratory for Infectious Disease Prevention and Control, and National Center for AIDS/STD Control and Prevention (NCAIDS), Chinese Center for Disease Control and Prevention, Beijing, China; Vanderbilt University, United States of America

## Abstract

**Background:**

Male circumcision (MC) has been shown to reduce the risk of female to male transmission of HIV. The goal of this survey was to explore the acceptability of MC among the Chinese and to identify factors associated with circumcision preference.

**Methods:**

A cross-sectional survey was conducted between September 2009 and December 2010. We interviewed 2,219 male community participants, from three high HIV prevalence provinces in western China. A structured questionnaire was used to collect data on MC knowledge, willingness to accept MC, reasons to accept or refuse MC, and sexual behaviors and health. For those who refused MC, a health education intervention providing information on the benefits of circumcision was conducted. We used multiple logistic regression models to identify factors associated with the acceptability of MC.

**Results:**

Of the respondents (n = 2,219), 44.6% (989/2,219) reported they would accept MC for the following reasons: promotion of female partners' hygiene (60.3%), redundant foreskin (59.4%), prevention of penile cancer (50.2%), enhanced sexual pleasure (41.4%), and protection against HIV and STDs (34.2%). The multivariable logistic regression showed that five factors were associated with MC willingness: long foreskin (OR = 15.98), residing in Xinjiang province (OR = 3.69), being younger than 25 (OR = 1.60), knowing hazards of redundant foreskin (OR = 1.78), and having a friend who underwent circumcision (OR = 1.36).

**Conclusion:**

The acceptability of male circumcision was high among the general population in China. Our study elucidates the factors associated with circumcision preference and suggests that more health education campaigns about positive health effects are necessary to increase the MC rate in China.

## Introduction

Randomized controlled trials (RCTs) in Africa have shown that male circumcision (MC) can reduce the risk of HIV infection by 50% to 60% in heterosexual men [1], [2], [3]. Several studies also suggested that uncircumcised men have higher risk of acquiring sexually transmitted diseases (STDs) including syphilis, gonorrhoea, and chlamydia than circumcised men [4], [5]. Improving access to MC may be the most effective way to prevent or control human immunodeficiency virus (HIV) transmission in high prevalence countries where transmission is predominantly heterosexual and MC is not generally practiced [6], [7].

China is facing a public health threat from HIV/AIDS with an estimated total of 740,000 people living with HIV/AIDS as of the end of 2009 [8]. Sexual transmission has accounted for more than half of the reported cases in China since 2007. For example, in 2007, the distribution was 44.7% for heterosexual transmission, 42.0% for injection drug users (IDU), 12.2% for men who have sex with men (MSM) [8], [9], [10]. The HIV epidemic in China has expanded from high risk groups to the general population [8] increasing the potential for heterosexual transmission. The AIDS epidemic is mainly concentrated in six provinces where the number of people with HIV/AIDS accounts for 83.0% of the total HIV/AIDS population: Yunnan, Guangxi, Henan, Xinjiang, Guangdong, and Sichuan. Four of these high HIV prevalence provinces are located in western China [9] where poverty is widespread, knowledge and risk perception of HIV are low, and unprotected commercial sexual encounters are common [11]. In Guangxi province, heterosexual transmission accounted for 60.9% of the reported HIV cases in 2009, followed by IDU (31.6%), MSM (0.8%), and others (6.7%). In Chongqing, in 2010, heterosexual transmission accounted for 39.5% of the reported cases, followed by IDUs (26.3%), MSM (15.4%), and others (18.8%). In Xinjiang, heterosexual transmission accounted for 41% of the reported cases in 2009, followed by IDU (37%) [9], [10]. Although the HIV prevalence in the general male population is low in these provinces (0.18% in Guangxi, 0.21% in Xinjiang, and 0.046% in Chongqing), evidence-based strategies are needed to contain the epidemic.

MC is not commonly practiced by the Chinese. While the prevalence of MC worldwide is almost 30%, only 5% of Chinese males are circumcised [12]. While the acceptability of MC has been studied in men and women in sub-Saharan Africa [13], [14], the United States [15], and Thailand [16], no population-level studies, with large sample size, prior to ours have been conducted in China, especially targeting general population. An earlier study assessed MC acceptability among MSM [17], which reported very low level of MC acceptability (16.9% would absolutely willing to participate and 26.4% would probably participate). Several other small scale studies, conducted by our group and published in Chinese journals explored MC acceptability among medial students (53.5% acceptability)[18], and migrant workers in coal mines (25.1% acceptability) [19], which were conducted by our group (published in Chinese) . Population-level data from diverse region with large sample size is needed to design and implement intervention strategies to promote circumcision as one of the HIV prevention tools. In this study we explored the acceptability of MC and documented the factors associated with circumcision preference among Chinese residents in Western China.

## Methods

### Study design and subjects

We conducted a cross-sectional survey using face-to-face structured interviews, in three western provinces (Guangxi, Chongqing and Xinjiang) of China. Three communities were conveniently selected in the capitals of three provinces. All adult male residents within the selected community were informed about the study and invited to participate in the interview during September 2009 to December 2010. The inclusion criteria for participation ere: age between 18–50 years, male residents, and residence in the target communities for at least three years. The exclusion criteria were individuals with hearing or speech impairment and those people who have undergone MC before.. Approval for this study was obtained from the Ethics and Human Subjects Committee of the Guangxi Medical University.

### Questionnaires and data management

A 51-item questionnaire was designed with the primary aim of obtaining information on the acceptability of male circumcision as an effective strategy to prevent HIV infection (i.e. “willingness to be circumcised”). The questionnaire had five sub-sections: demographic characteristics, general knowledge about AIDS and MC, acceptability of circumcision, reasons to accept or refuse MC, and sexual behaviors and sexual health. Few open-ended questions were asked, and most primary outcome variables were assessed by asking Yes/No questions, such as “Can MC prevent STDs?” Smoking habit was defined as smoking (smoking at least one cigarette daily for more than three months), no smoking (never or former smoking with cessation for more than 3 months). Drinking habit was defined as current drinking (drinking at least once each week for more than 3 months) and no drinking (never or former drinking). Condom use was defined as consistent (always use), and inconsistent use (use only sometimes or occasionally), and never use.

To assess knowledge about AIDS and MC, we asked twenty questions, including ten questions about general knowledge of AIDS such as the pathway of HIV transmission and infection, and eleven questions about MC including the most suitable period and targeted population, and the advantages or adverse effects after surgery [17]. For AIDS knowledge we computed the average score among all interview subjects; each correct answer was given a score. Willingness to accept circumcision was assessed with the question “ do you want to be circumcised ?”, and the response categories were “definitely willing”, “probably willing”, “definitely not willing” and “probably not willing”. For analysis, we dichotomized the groups of “definitely willing” and “probably willing” into a single variable of “willingness to accept MC”, and the groups of “definitely not willing” and “probably not willing” as “refusal to accept”. To assess reasons to accept or refuse circumcision, we also asked eleven open-ended questions, which enquired about the advantages and disadvantages of MC and surgery cost.

For those who classified as refusing MC, a health education intervention, including the explanation of the benefits of MC and knowledge of circumcision was carried out. The contents of the intervention included information about how MC reduces HIV and STDs, improves penile hygiene, has few surgical complications which could be easily managed, and is available free of charge.

All data were collected by trained research Assistants (RAs). After the subjects provided their written informed consent to participate in the study, RAs conducted the detailed interviews following the structured guidelines.

### Analysis

All the data were entered into EpiData software (EpiData 3.0 for Windows; The EpiData Association Odense, Denmark) and analyzed using SPSS for Windows Version 14.0 (SPSS, Chicago, IL, USA). Descriptive statistics was generated for each of the variables corresponding to specific questions in the survey, including general characteristics and reasons to accept or refuse MC. To compare basic characteristics between two groups, we used chi-square tests. Multivariate logistic regression analysis was performed to identify factors associated with the acceptability of MC. Included in the model were variables that showed a statistically significant association (*P*<0.05) with the willingness to be circumcised in the bi-variate analyses. All statistical tests were two-sided with a significant level of *P*<0.05.

## Results

### Demographic characteristics

A total of 2325 subjects participated in the interview and 2219 completed the whole interview (Completion rate: 95%). As shown in Table 1, of the 2219 respondents 30.1% (n = 668) were from Guangxi; 33.9% (n = 753) from Chongqing; and 36.0% (n = 798) from Xinjiang. Over half (56%) were aged 35 or below; 59.4% were married; 73.0% had a high school education or above; and 80.2% had stable jobs. Most of the respondents (81.4%, n = 1807) had sexual intercourse in the past three months. Of those,, 49.6% used condoms inconsistently and 27.8% had never used a condom.

**Table 1:** Demographic characteristics of 2219 men interviewed in three provinces, western region of China, 2010.

Variables	No.	Percent
Total samples	2219	100.0
Provinces		
Guangxi	668	30.1
Chongqing	753	33.9
Xinjiang	798	36.0
Age		
18–25	549	24.7
25–35	685	30.9
Over 35	985	44.4
Marital status		
Married	1319	59.4
Never married	858	38.7
Divorced/separated/widowed	42	1.9
Education level		
Junior school or below	600	27.0
High school or above	1619	73.0
Employment status		
Employed	1779	80.2
Unemployed	440	19.8
Smoking		
Yes	1053	47.5
No	1166	52.5
Drinking		
Yes	741	33.4
No	1478	1478
Had sexual intercourse in the past 3 months		
Yes	1807	81.4
No	412	18.6
Condom use		
Consistent use	407	22.6
Inconsistent use	897	49.6
Never use	503	27.8

### Acceptability of MC and Reasons to accept or refuse MC

Almost half of respondents (44.6%) reported willingness to accept circumcision to prevent HIV and STDs infection. As shown in Table 2, of willing to accept MC (n = 989), 60.3% thought it would improve sexual partners' hygiene; 59.4% were willing to remove redundant foreskin; 50% thought saw MC as a way to prevent penile cancer; and 34.2% believed MC would prevent HIV and STDs. Of those who refused MC (n = 1230), the majority (81.1%) reported that it would not be effective for them and 10.4% were worried about the reduction of sexual ability.

**Table 2:** Reasons to be accept or refuse MC among the Chinese men.

	Frequency	Percent
**Willing to accept MC**	989	100.0
Improve female partners' hygiene	596	60.3
Redundant foreskin	587	59.4
Prevention of penile cancer	496	50.2
Protection against HIV and STDs	338	34.2
Enhance sexual pleasure	409	41.4
Better penile appearance	109	11.0
Traditional or religious reason	38	3.8
**Refuse MC**	1230	100.0
Not necessary or not effective	997	81.1
Concern about potential danger associated with surgery	162	13.2
Concern about reducing sexual ability	128	10.4
Concern about expensive surgery cost	70	5.7

### Knowledge about MC

The willingness-to-be-circumcised group (WTC) possessed better knowledge about AIDS and MC than the non-WTC group. For example, 44.3% WTC subjects knew that MC can prevent penile inflammation and cancer, compared to 31.5% non-WTC(*P*<0.05). Overall, 30% knew that MC can prevent AIDS and STDs, but more subjects in the WTC group had this knowledge (25.7% versus 18.1%, *P*<0.05). More WTC subjects knew about the hazards of redundant foreskin (90.1% versus 77.8%, *P*<0.05), and more had friends who ever had circumcision (62.7% versus 39.1%, *P*<0.05).

### Effects of health education interventions for those refusing MC

To explore reasons for not accepting MC and estimate the effects of health education intervention and measures, we explained the benefits and related knowledge of circumcision to 1230 subjects who were not willing to be circumcised. Interestingly, when they were told that MC would reduce HIV and STDs, 40.7% (500/1230) of subjects changed their views and were willing to accept MC. When we explained that MC had only very low surgery-related complications, another 21.1% (259/1230) were willing to accept MC. When we explained that MC could be arranged free of charge, 20.5% (252/1230) said they would accept circumcision. However, 58.9% (725/1230) still refused MC.

### Factors associated with willingness to accept MC

We carried out univariate analysis to explore potential factors associated with circumcision preferences. Table 3 and Table S1 showed that seventeen potential factors were taken into account and all, except smoking and drinking, were significantly associated with the willingness to be circumcised. A multivariable logistic regression model identified five factors associated with WTC (Table 4): redundant or too-long foreskin (OR = 15.98), residence in Xinjiang province (OR = 3.69), age below 25 (OR = 1.60), knowledge about the hazards of redundant foreskin (OR = 1.78), and having a circumcised friend (OR = 1.36).

**Table 3:** Factors associated with the willingness to be circumcised.

Variables	WTC groupn (%)	Non-WTC groupn (%)	χ^2^	*P* value
Can MC prevent penile inflammation and cancer?			38.09	0.000
Yes	438(44.3)	388(31.5)		
No	551(55.7)	842(68.5)		
Can MC prevent AIDS and STDs?				
Yes	254(25.7)	223(18.1)	18.53	0.000
No	735(74.3)	1007(81.9)		
Can MC improve sexual partners' hygiene?			53.55	0.000
Yes	540(54.6)	480(39.0)		
No	449(45.4)	750(61.0)		
Can MC enhance sexual pleasure in the future?			29.92	0.000
Yes	356(36.0)	311(25.3)		
No	633(64.0)	919(74.7)		
Can MC improve penile appearance?			7.41	0.006
Yes	620(10.4)	88(7.2)		
No	369(89.6)	1142(92.8)		
Can redundant foreskin be hazardous?			59.43	0.000
Yes	891(90.1)	957(77.8)		
No	98(9.9)	273(22.2)		
Whether or not your friends underwent MC			121.97	0.000
Yes	620(62.7)	481(39.1)		
No	369(37.3)	749(60.9)		
Do you feel that your foreskin is redundant or too long?				
Yes	440(44.5)	50(4.1)	520.66	0.000
No	549(55.5)	1180(95.9)		
Ever had sexual intercourse?			5.51	0.019
Yes	784(79.3)	1023(83.2)		
No	205(20.7)	207(16.8)		

**Table 4:** Multivariate analysis of willingness to be circumcised among 2219 men interviewed.

Variables	Adjusted OR (95% CI)	P value
Province		
Guangxi	1.00	
Chongqing	2.46(1.86∼3.25)	0.000
Xinjiang	3.69(2.78∼4.91)	0.000
Age		
Over 35	1.00	
25–35	1.01(0.82∼1.22)	0.950
18–25	1.60 (1.30∼1.98)	0.000
Can redundant foreskin be hazardous?		
No	1.00	
Yes	1.78(1.31∼2.43)	0.000
Whether or not your friends underwent MC		
No	1.00	
Yes	1.36(1.10∼1.70)	0.006
Do you feel that your foreskin is redundant or too long?		
No	1.00	
Yes	15.98(11.62∼21.99)	0.000

## Discussion

Our survey showed a high level of MC acceptability (44.6%), but this proportion is lower than the reported median acceptability rate (62%) in sub-Saharan Africa with 29–81%14]. The main associated factors included long foreskin, residing in Xinjiang province, age below 25 years, and the influence of circumcised friends Protection against HIV and STDs was not included as a reason for willingness to be circumcised. Several overseas studies also reported partially similar factors determining MC acceptability, including maintaining penile hygiene, receiving free medical care, increasing sexual pleasure, and protection against HIV infection [14], [17], [20].

Overall, the average MC rate worldwide, among non-muslims, is estimated at 30–34%, and the practice is very common in West Africa, parts of central and Eastern Africa, the United States, and Republic of Korea [21]. However, in China, MC is not a common practice; less than 5% males are circumcised and many of these procedures were carried to alleviate medical complaints such as tight foreskin [12]. Our results show that overall MC acceptability is high, especially among residents in Xinjiang province (OR = 3.69) where many Muslims who are circumcised as infants for religious reasons live. It is possible that the universal practice among Muslims influenced greater MC acceptability among the population in other groups across Xinjiang province. By residing in the same community with other Muslims, these uncircumcised men may be more aware of the procedure and be likely to have heard more positive references from their friends or others who are circumcised.

In the logistic regression model, we found that young men below 25 years were more willing to accept MC than men over 35 years. Our findings are consistent with the findings in the Dominican Republic and Kenya [22], [23]. This high acceptability among younger male may be due to the fact that young men are more knowledgeable about MC and aware of the protection against that it confers. In this study we found that more young persons knew that MC can prevent penile inflammation and cancer (data not shown). Moreover, they also paid more attention to their sexual hygiene and sexual health.

Consistent with the findings in other studies [23], [24], we found improved MC acceptability among men who had previously refused after providing information about the benefits. In the non-WTC group, most (81.1%) considered that MC was not necessary or not effective; however, after the subjects in this group were provided with information about the benefits(including “increasing genital hygiene”, “reducing cancer”, “reducing HIV and STDs infection”, and few surgery complications), more uncircumcised men changed their position and reported greater willingness to be circumcised. This suggests that appropriate education campaigns are necessary if we are to promote MC's acceptability among the Chinese.

We missed collecting information about the number of sexual partners in the current study, which could have important public health implications. It is not clear whether a high proportion of those who accepted MC are those who have multiple sex partners. However, in western China, data from the AIDS prevention and control system shows that HIV epidemic in female population is aggravating with more reported HIV infection among pregnant women and female commercial sex workers. Also, in recent years, high-risk heterosexual behaviors are more common among HIV infected males in these regions. This suggests that general male population from these three provinces may be are at high risk to infect others through heterosexual transmission.

Our study has several limitations. First, we used convenience sampling to extrapolate results to the general population in the three western provinces in China, which may lead to selection bias. Those who participated in the study were perhaps more concerned about their health and more interested in the topic. However, the population characteristics of our selected communities were similar to those in other communities in the region. To have a wider picture from the whole region, another study involving more communities and a larger sample size is necessary. Additionally, all data collected was based on self-reported behaviors and characteristics (e.g., self-reported long foreskin) without clinical examination or other confirmation. Finally, although HIV epidemic in China is expanding from high risk groups to the general population, the average prevalence of HIV was still low among Chinese general population, which may partially explain why many people are willing to accept MC but have not undergone the circumcision yet.

In summary, the findings of this study show that the acceptability of male circumcision is high among the general population in western China, though lower than many African countries. The factors identified as predictors of willingness to undergo MC can be used to design programs to promote MC among the Chinese. The educational information session about the benefits of MC identified some preliminary data, which can guide future educational campaigns. However, further studies are needed to assess the feasibility and effectiveness of any educational campaign with a rigorous evaluation component in place.

## Supporting Information

Table S1 General factors associated with the willingness to be circumcised.
(DOC)

## References

[1] Auvert B, Taljaard D, Lagarde E, Sobngwi-Tambekou J, Sitta R (2005). Randomized, controlled intervention trial of male circumcision for reduction of HIV infection risk: the ANRS 1265 Trial.. PLoS Med.

[2] Bailey RC, Moses S, Parker CB, Agot K, Maclean I (2007). Male circumcision for HIV prevention in young men in Kisumu, Kenya: a randomised controlled trial.. Lancet.

[3] Gray RH, Kigozi G, Serwadda D, Makumbi F, Watya S (2007). Male circumcision for HIV prevention in men in Rakai, Uganda: a randomised trial.. Lancet.

[4] Diseker RA, Peterman TA, Kamb ML, Kent C, Zenilman JM (2000). Circumcision and STD in the United States: cross sectional and cohort analyses.. Sex Transm Infect.

[5] Weiss HA, Thomas SL, Munabi SK, Hayes RJ (2006). Male circumcision and risk of syphilis, chancroid, and genital herpes: a systematic review and meta-analysis.. Sex Transm Infect.

[6] Williams BG, Lloyd-Smith JO, Gouws E, Hankins C, Getz WM (2006). The potential impact of male circumcision on HIV in Sub-Saharan Africa.. PLoS Med.

[7] Weiss HA, Quigley MA, Hayes RJ (2000). Male circumcision and risk of HIV infection in sub-Saharan Africa: a systematic review and meta-analysis.. Aids.

[8] Teng T, Shao Y (2011). Scientific approaches to AIDS prevention and control in china.. Adv Dent Res.

[9] Chinese Ministry of Health, Joint United Nations Programme on HIV/AIDS, World Health Organization (2010). 2009 Update on the HIV/AIDS Epidemic and Response in China..

[10] Chinese Ministry of Health, Joint United Nations Programme on HIV/AIDS, World Health Organization (2008). 2007 Update on the HIV/AIDS Epidemic and Response in China..

[11] Shao-Ru Z, Hong Y, Xiao-Hong L, Jian-Ping P, Wan-Xia Y (2010). The personal experiences of HIV/AIDS patients in rural areas of western China.. AIDS Patient Care STDS.

[12] Ben KL, Xu JC, Lu L, Lu NQ, Cheng Y (2009). [Male circumcision is an effective “surgical vaccine” for HIV prevention and reproductive health].. Zhonghua Nan Ke Xue.

[13] Gust DA, Kretsinger K, Gaul Z, Pals S, Heffelfinger JD (2011). Acceptability of Newborn Circumcision to Prevent HIV Infection in the United States.. Sex Transm Dis.

[14] Herman-Roloff A, Otieno N, Agot K, Ndinya-Achola J, Bailey RC (2011). Acceptability of medical male circumcision among uncircumcised men in kenya one year after the launch of the national male circumcision program.. PLoS One.

[15] Westercamp N, Bailey RC (2007). Acceptability of male circumcision for prevention of HIV/AIDS in sub-Saharan Africa: a review.. AIDS Behav.

[16] Tieu HV, Phanuphak N, Ananworanich J, Vatanparast R, Jadwattanakul T (2010). Acceptability of male circumcision for the prevention of HIV among high-risk heterosexual men in Thailand.. Sex Transm Dis.

[17] Ruan Y, Qian HZ, Li D, Shi W, Li Q (2009). Willingness to be circumcised for preventing HIV among Chinese men who have sex with men.. AIDS Patient Care STDS.

[18] Deng W, Yang XB, Jiang JJ, Liang BY, Lu ZP (2010). The Influential factors for the willingness to circumcision among medical male college students in Guangxi.. Modern Preventive Medicine.

[19] Jiang JJ, Liang X, Yang XB, Deng W, Wei B (2011). Acceptability and related factors of male circumcision among farmer workers in coalmine.. Chin J Public Health.

[20] Weiss HA, Halperin D, Bailey RC, Hayes RJ, Schmid G (2008). Male circumcision for HIV prevention: from evidence to action?. Aids.

[21] UNAIDS (2007). Male circumcision: global trends and determinants of prevalence, safety and acceptability..

[22] Mattson CL, Bailey RC, Muga R, Poulussen R, Onyango T (2005). Acceptability of male circumcision and predictors of circumcision preference among men and women in Nyanza Province, Kenya.. AIDS Care.

[23] Brito MO, Caso LM, Balbuena H, Bailey RC (2009). Acceptability of male circumcision for the prevention of HIV/AIDS in the Dominican Republic.. PLoS One.

[24] Kebaabetswe P, Lockman S, Mogwe S, Mandevu R, Thior I (2003). Male circumcision: an acceptable strategy for HIV prevention in Botswana.. Sex Transm Infect.

