# Supplementary material for: Factors Influencing Chinese Male's Willingness to Undergo Circumcision: A Cross-Sectional Study in Western China
Source: PLoS One. 2012 Jan 12;7(1):e30198. doi: 10.1371/journal.pone.0030198 (PMC3257276; doi:10.1371/journal.pone.0030198)
Supplement: Table S1 — General factors associated with the willingness to be circumcised. (DOC) [file pone.0030198.s001.doc]

**Supporting information**

**Table S1** General factors associated with the willingness to be circumcised

**Table S1** General factors associated with the willingness to be circumcised

| Variables | WTC group  n (%) | Non-WTC group  n (%) | χ^2^ | *P* value |
| --- | --- | --- | --- | --- |
| Province |  |  | 203.67 | 0.000 |
| Guangxi | 155(15.7) | 513(41.7) |  |  |
| Chongqing | 354(35.8) | 399(32.4) |  |  |
| Xinjiang | 480(48.5) | 318(25.9) |  |  |
| Age |  |  | 22.87 | 0.000 |
| 18-25 | 293(29.6) | 256(20.8) |  |  |
| 25-35 | 286(28.9) | 399(32.4) |  |  |
| Over 35 | 416(41.5) | 575(46.7) |  |  |
| Marital status |  |  | 28.38 | 0.000 |
| Married | 541(65.8) | 809(54.7) |  |  |
| Never married | 425(43.0) | 402(32.7) |  |  |
| Divorced/separated/widowed | 23(2.3) | 19(1.5) |  |  |
| Education level |  |  | 45.85 | 0.000 |
| Junior school or below | 197（19.9） | 403（32.8） |  |  |
| High school or above | 792（80.1） | 827（67.2） |  |  |
| Employment status |  |  | 7.82 | 0.005 |
| Employed | 819（82.8） | 960（78.0） |  |  |
| Unemployed | 170（17.2） | 270（22.0） |  |  |
| Smoking |  |  | 0.08 | 0.777 |
| Yes | 466（47.1） | 587（47.7） |  |  |
| No | 523（52.9） | 643（52.3） |  |  |
| Drinking |  |  | 0.12 | 0.735 |
| Yes | 334（33.8） | 407（33.1） |  |  |
| No | 655（66.2） | 823（66.9） |  |  |
| AIDS knowledge |  |  |  |  |
| Above average score | 445 (45.0) | 600 (48.8) | 3.15 | 0.076 |
| Below average score | 544 (55.0) | 630 (51.2) |  |  |

Note: the average score is 8.04 for all interviewed men.
